# Vesicular transport in the autophagic route: RAB GTPases as pivotal regulators of autophagy

**DOI:** 10.1042/BCJ20253092

**Published:** 2025-10-17

**Authors:** Romina Abba, María Isabel Colombo

**Affiliations:** Facultad de Ciencias Médicas, Laboratorio de Mecanismos Moleculares Implicados en el Tráfico Vesicular y la Autofagia-Instituto de Histología y Embriología (IHEM)- Universidad Nacional de Cuyo, CONICET

**Keywords:** autophagy, membrane traffic, RAB GTPases

## Abstract

Autophagy is recognized as one of the two main intracellular recycling pathways that play an essential role in cellular homeostasis by maintaining accurate energy levels and carrying out quality control functions. One of the major autophagic mechanisms, the so-called macroautophagy, is involved in the lysosomal degradation of different cytoplasmic components, such as long-lived proteins and damaged or dysfunctional organelles. Numerous studies have demonstrated that participation of intracellular membrane trafficking events is key for the progression of autophagy. In this review, we will focus on the small GTPases of the RAS-related in brain protein family, which have a crucial role in vesicular transport.

## Autophagy

Autophagy is a self-degradation process conserved among eukaryotes that is mainly activated in response to the cell’s nutritional status as well as stress conditions, maintaining an adequate cellular metabolism [1]. In mammalian cells, three major autophagy mechanisms known as macroautophagy, microautophagy, and chaperone-mediated autophagy have been defined, which differ in both morphological and mechanistic features. Numerous studies have also shown that although these processes share some common molecular components, they use several specific factors to perform their function [2]. *Autophagy-related* genes (*ATGs*) encode for most of the core proteins with a critical role in autophagosome biogenesis, the hallmark of macroautophagy (hereafter autophagy) [3–8]. Three major steps characterize autophagy: (i) phagophore or isolation membrane development; (ii) autophagosome formation; (iii) autolysosome generation. The first step involves the generation of a transient flattened cisterna known as the phagophore or isolation membrane involved in the sequestration of different components destined for degradation. Afterward, this structure elongates and closes, forming the double-membrane autophagosome, which finally fuses with late endosomes and the lysosomes (i.e. maturation) to degrade the engulfed materials and recycle the resulting metabolites [9].

The core ATG machinery is subdivided into six functional complexes, which orchestrate the formation of the phagophore and the biogenesis of autophagosomes [4,10,11]. These key complexes are the Unc-51-like autophagy-activating kinase (ULK) kinase complex, the ATG9A-positive vesicles, the class III phosphatidylinositol 3-kinase complex I (PI3KC3-C1), the ATG2 proteins-WIPI4 complexes, and the ubiquitin-like ATG12 and LC3 conjugation systems [11,12]. The initial complex that leads to the induction of autophagosome formation is the ULK kinase complex, which is composed of ULK1 or ULK2, FIP200 (focal adhesion kinase family interacting protein of 200 kD), ATG13, and ATG101. Mechanistic target of rapamycin complex 1 (mTORC1) is a crucial negative regulator of autophagy in response to nutrient availability. Under full nutrient conditions, mTORC1 is active and directly phosphorylates ULK1 and ATG13, suppressing autophagy induction [13]. In contrast, 5′-adenosine monophosphate-activated protein kinase (AMPK) promotes autophagy by phosphorylating and activating ULK1 [14,15]. Phagophore formation is also promoted by PI3KC3-C1, consisting of VPS34, VPS15, BECLIN 1 (BECN1), ATG14, and nuclear receptor-binding factor 2, which generates phosphatidylinositol 3-phosphate (PI3P), a crucial lipid for the association of the subsequent complexes mentioned below. This lipid is essential for the initial step in autophagosome formation, the generation of the so-called omegasomes [5,16], cup-shaped regions from the endoplasmic reticulum (ER) characterized by the presence of the protein double-FYVE containing protein 1 (DFCP1), whose ATPase activity is critical for the final stages of omegasome constriction that leads to autophagosome biogenesis [17,18]. The ATG9A vesicles, which are present in the vicinity of phagophores, provide membranes for phagophore expansion. ATG9 has a phospholipid scrambling activity, and this activity is required for the supply of phospholipids to the expanding autophagosome [19,20]. In the yeast *Saccharomyces cerevisiae,* Atg9 facilitates the recruitment of PI3KC3-C1, which is essential for Ptdlns3P production, to the phagophore assembly site known as the pre-autophagosomal structure (PAS).

The ATG2 proteins-WIPI4 (WD repeat domain phosphoinositide-interacting protein 4) complexes, composed of ATG2A or ATG2B and WIPI4, contribute to the expansion of the phagophore by transferring lipids mostly from the ER. The formation of the mature autophagosome depends on the two ubiquitin-like conjugation systems, which lead to the final closure of the phagophore, making it fusion competent. The ATG12-ATG5-ATG16L1 complex, where ATG12 is covalently linked to ATG5, forming a conjugate that interacts with ATG16L1, is essential for guiding the conjugation of LC3 (microtubule-associated protein 1 light chain 3) proteins through the second ubiquitin-like conjugation system to the phosphatidylethanolamine present in the phagophore membrane. LC3 proteins are one of the best studied and critical ATG proteins recruited to the autophagosomal membranes from the first steps and associate with the forming phagophore to both the external and internal membranes [21]. This particular ATG, unlike others, remains associated with the autophagic membranes during the whole maturation process of the pathway to the final fusion with the lysosomes. LC3, like the ATGs, is one of the members of a family of proteins involved in both selective and non-selective degradation processes [9,22]. In selective autophagy, selective autophagy receptors (SARs) mediate the binding of the pool of LC3 protein in the interior of the phagophore to specific cargoes [23]. Binding to SARs is mediated via short peptides such as the LC3-interacting region, also known as ATG8-interacting motifs, or the ubiquitin-interacting motif-like sequence [23,24]. Some SARs, such as nuclear dot protein 52 kDa (NDP52/CALCOCO2), sequestosome 1 (SQSTM1/p62), and optineurin, are soluble proteins and bind to ubiquitinylated cargoes, whereas others are part of complexes or inserted into the limiting membrane of organelles. By simultaneously binding the cargo and LC3, SARs mediate the sequestration of the cargo into nascent autophagosomes.

The molecular mechanism of autophagy and the function of the ATGs in the different steps of this process have been extensively described in comprehensive reviews [1,3,5,6,11]. In this review, we will focus on those small GTPases of the RAS-related in brain (RAB) protein family that are normally involved in vesicular transport but have also been associated with autophagy.

## Vesicular trafficking and autophagy

One of the hallmarks of eukaryotic cells is their extensive system of endomembranes, which constitute a network of intracellular compartments that communicate and exchange cargo molecules. Over the last 30 years, a comprehensive overview of the intermediate structures involved in autophagy and their relationship with other intracellular organelles such as the ER, the Golgi apparatus, mitochondria, and components of the endocytic system has emerged, but it is still an area of active research since numerous questions remain to be answered [25–27]. Importantly, alterations in this functional cross-talk are in several cases the cause of human diseases, and some of them have been associated with dysfunction of specific members of the RAB family of small GTPases (see below).

## RABs as one of the main molecular co-ordinators of vesicular transport

**Figure 1:**
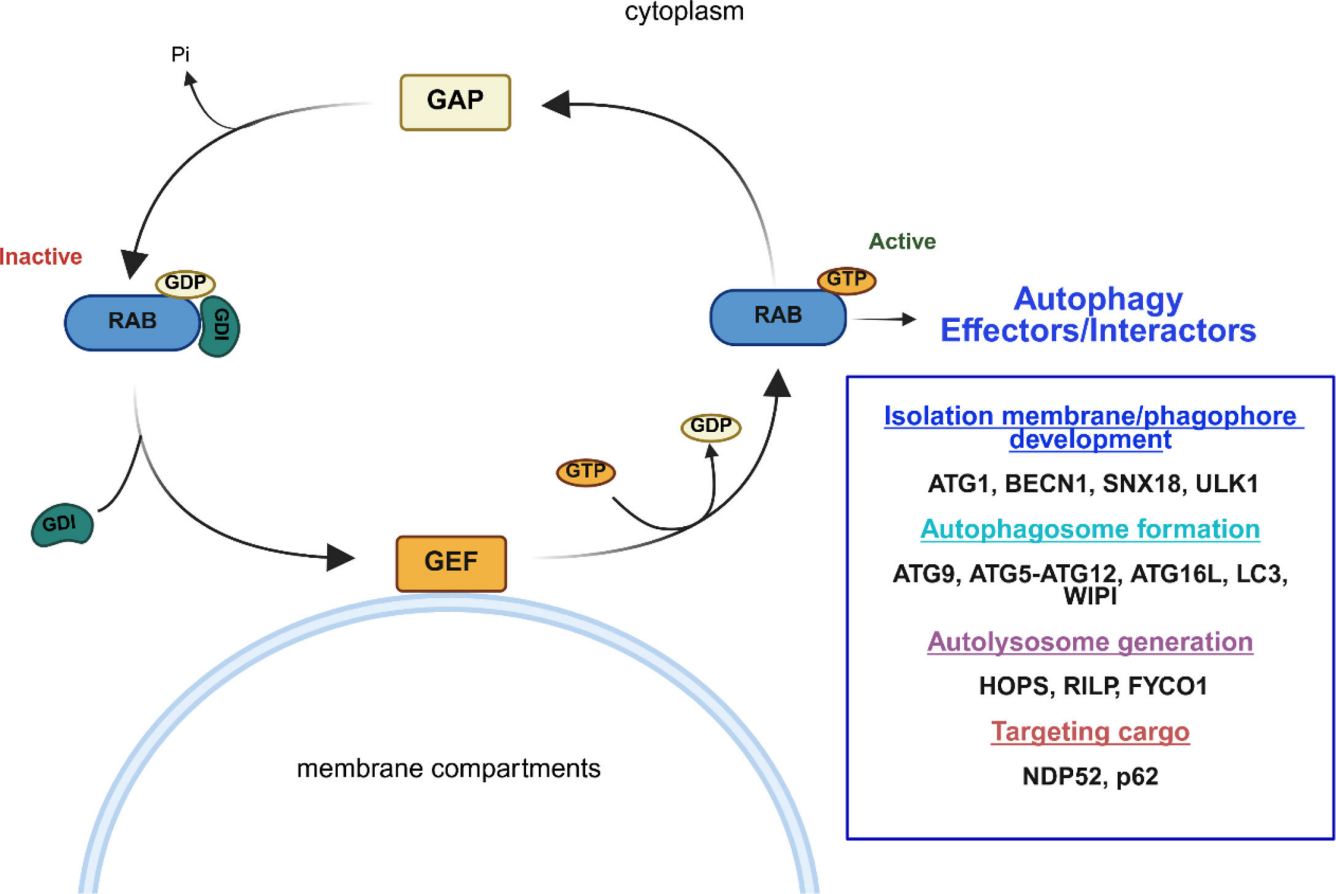
RAB proteins function as molecular switches, alternating between inactive GDP-bound conformations and active GTP-bound conformations. This alternating cycle, regulated by guanine nucleotide exchange factors (GEFs) and GTPase-activating proteins (GAPs), facilitates dynamic interactions with membranes and the recruitment of the machinery necessary for the initiation of autophagy, such as ULK1. Additionally, they participate in the formation of autophagosomes through direct effectors such as ATGs and LC3, as well as in the fusion of lysosomes with autophagosomes through proteins like RILP and HOPS, among direct mediators. HOPS, homotypic fusion and protein sorting; RILP, Rab-interacting lysosomal protein.

The RAB comprises a large family of small GTP-binding proteins [28–30]. These GTPases have a molecular weight that varies between 22 and 27 kDa. RABs with more than 60 members in the human genome [31]. In concert with the soluble NSF attachment protein receptors proteins (SNAREs) and tethering factors, RABs are master regulators of intracellular vesicle transport, since they participate in many events such as vesicle budding, transport, tethering, and fusion with the target compartment. RABs are distributed to different membranous compartments, sometimes to specific microdomains, and provide identity with these subcellular compartments. In addition, these proteins have also been associated with specific membrane contact sites between different organelles [32], modulating distinct events such as lipid transport and mitochondrial fission or mitophagy [32–35]. Upon regulated association to a compartment, RABs act as molecular switches by recruiting diverse effector molecules through direct binding. RABs can convert from an inactive GDP-bound conformation to an active, membrane-associated GTP-bound form, and vice versa. The switch to the GTP-bound form depends on the interaction with specific guanine nucleotide exchange factors (GEFs), whereas the hydrolysis of the GTP bound to RABs is stimulated by enzymes called GTPase-activating proteins (GAPs) [36]. GTP association and subsequent GTP hydrolysis allow a very dynamic and regulated membrane binding and release, respectively, of RABs (see Figure 1). Importantly, numerous RAB proteins implicated in vesicular transport have also been shown to be involved in different steps of the autophagy pathway [5,37–40].

## RABs in autophagy

RABs participate in distinct steps of autophagy, and proteomic analysis has demonstrated that several ATG proteins interact with several of them, highlighting that a number of ATG proteins are RAB effector molecules (please see Figure 1) [41].

### RAB GTPases involved in the initial stages of the autophagic pathway

Some RAB proteins are key in the initial steps of autophagy, participating in the nascent autophagic vesicle development, omegasome formation, favoring conjugation of critical autophagic proteins, or even in upstream signaling events such as mTOR regulation.

**Figure 2:**
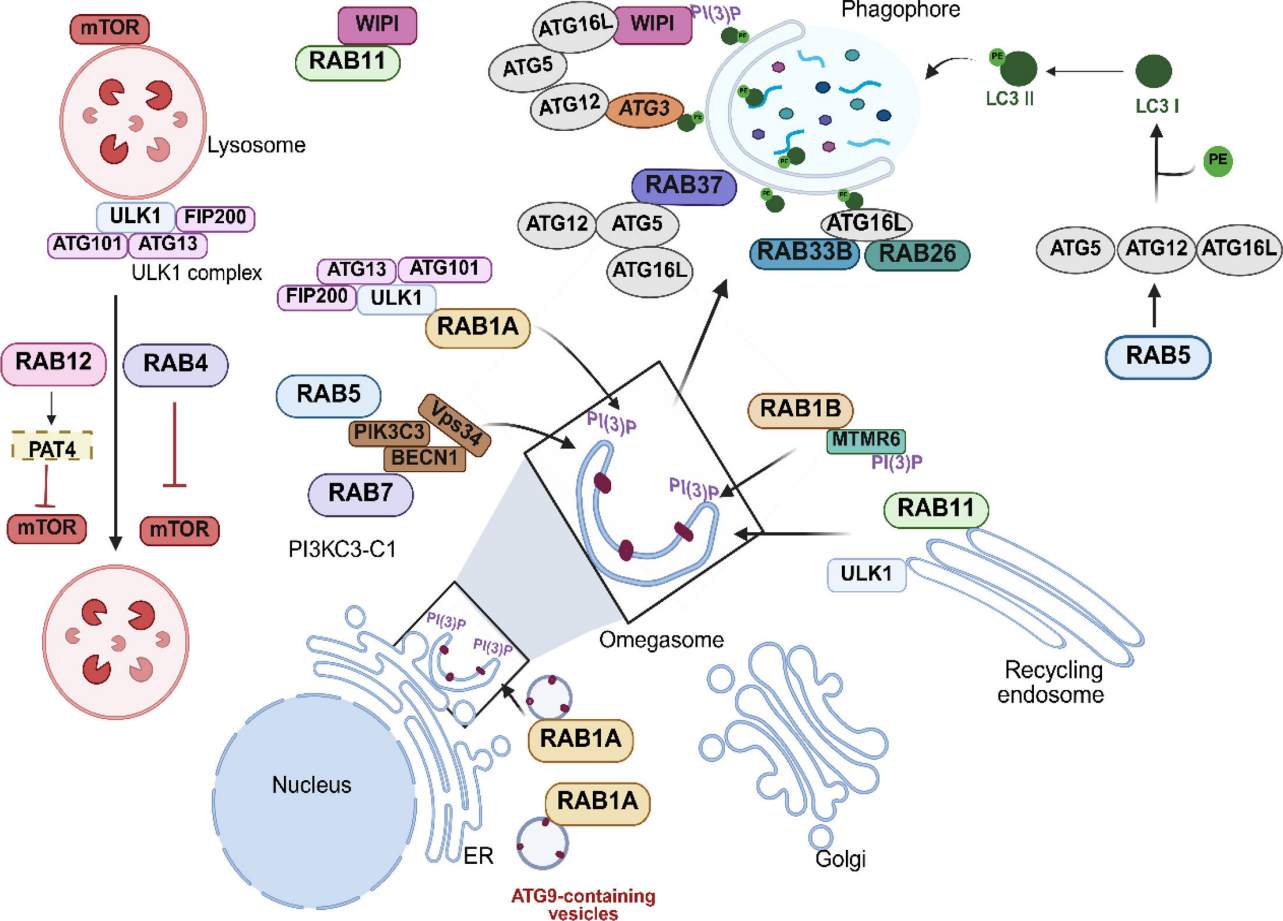
Some RAB GTPases are pivotal in autophagy initiation, omegasome formation, and nascent autophagic vesicle development, as well as in negatively regulating the mTOR complex. RAB1A, RAB5, RAB7, and RAB11 are very important for the initial steps of autophagy by interacting with critical autophagy proteins such as ULK1, the PI3KC3-C1 complex, WIPI, and different essential ATGs. These Rabs help make omegasomes and early autophagic vesicles (phagophores). Additionally, certain Rab proteins, including RAB1A, RAB12, and RAB4, regulate mTOR by inhibiting its activity, thereby promoting autophagy activation.

RAB1 localizes to the ER-Golgi intermediate compartment and cis Golgi, and modulates the transport from the ER to the Golgi, as well as retrograde transport inter Golgi cisternae and back to the ER [42]. This RAB presents two highly conserved isoforms, RAB1A and RAB1B. We have previously shown, by knocking down RAB1B and by overexpressing RAB1B N122I, a nucleotide-free and negative dominant mutant, that RAB1B is involved in autophagosome formation at ER exit sites, suggesting that trafficking from the ER may be critical for the initial steps of autophagy [43]. These data are consistent with results obtained in yeast in which deletion of *TRS85*, a specific subunit of the TRAPPIII complex that is one of the GEFs of Ypt1/RAB1 and also localizes to the PAS, is critical for the generation of the PAS through the recruitment of Ypt1, which in turn interacts with components of the ATG machinery such as ATG1, ATG11, ATG17, ATG23, and the PI3KC3-C1 [19,44–47]. Interestingly, it was shown that RAB1B participates in autophagosome formation by also modulating the levels of Ptdlns3P at the omegasome via the interaction with the myotubularin-related protein 6 phosphatase [48]. Furthermore, it has been recently shown that RAB1A, in its GTP-active conformation, binds and recruits PI3KC3-C1, thus modulating the levels of Ptdlns3P [49]. Consistently, depletion of RAB1 leads to a decrease in the formation of DFCP1, a PI3P-binding protein enriched in the omegasomes, as mentioned before [5,16]. Also, it was recently shown that in yeast, Ypt1, the RAB1 ortholog, is phosphorylated by TOR at the Ser174 residue, and the Ypt1*^S174D^* phosphorylation mimic mutant prevents both PAS recruitment and activation of ATG1, indicating that Ypt1 functions as an assembly factor required for the initiation of autophagy [50]. Furthermore, the activity of RAB1A is required for the transport of ATG9A-containing vesicles and interacts with the ULK kinase complex at the phagophore (see Figure 2) [47,51].

RAB4 overexpression blocks mTORC1, and this leads to the formation of LC3-positive vesicles, possibly autophagosomes. In addition, overexpressed RAB4 localizes on mitochondria during autophagy induced by nutrient starvation or treatment with rapamycin and provokes an accumulation of mitochondria, likely by inhibiting mitophagy. Furthermore, overexpression of RAB4^Q72L^, a dominant-positive mutant deficient in its GTPase activity, causes the formation of mitochondrial tubular networks upon starvation [52,53]. Taken together, these findings suggest that RAB4 has a role in both autophagy and mitochondrial homeostasis maintenance (please see Figure 2). It has also been shown that the early endosomal. RAB5 controls both the localization and activation of mTORC1 in mammalian cells and in yeast [54]. In addition, as depicted in Figure 2, RAB5 is part of a signaling network that activates the PI3KC3-C1 complex, promoting ATG12 conjugation to ATG5, leading to the elongation of the phagophore. Interestingly, RAB5 initiates autophagy in a starvation-independent manner but in response to growth factor shortages [54–56]. As shown in Figure 2, RAB37 has also been implicated in the early stages of autophagy by localizing to the isolation membrane, where it interacts with ATG5, facilitating the assembly of the ATG5-ATG12-ATG16L1 complex [57].

RAB6 localizes to the Golgi, where it mediates the retrograde transport between the Golgi and from this organelle to the ER and endosomes [58]. It has been shown that Ypt6, the yeast ortholog of RAB6, is essential for autophagy initiation [59,60]. An additional report has shown that RAB6 acts as a regulator of mTORC1 and autolysosome activity [61]. In particular, depletion of RAB6 in *Drosophila* leads to the accumulation of enlarged autophagic vacuoles due to a decrease in the transport of lysosomal enzymes to the autolysosomes [61]. Thus, this RAB, like others described below, seems to have a dual function, not only at the initial steps of the pathway by modulating mTORC1 activity but also participating in autophagy maturation, likely by an indirect mechanism (i.e. transport of degradative enzymes) [61].

Another RAB involved in mTORC1 regulation is RAB12. Depletion of this RAB leads to an activation of mTORC1, leading to autophagy inhibition [62]. The activation of mTORC1 is connected to a block in the degradation of the proton-coupled amino acid transporter 4 (PAT4) in RAB12-depleted cells, which accumulates PAT4 at the plasma membrane, increasing amino acid uptake and thus activating mTORC1 (see Figure 2) [62]. A very recent publication indicates a critical role for RAB12 in a degenerative disease of the retinal pigment epithelium and choroid.

**Figure 3:**
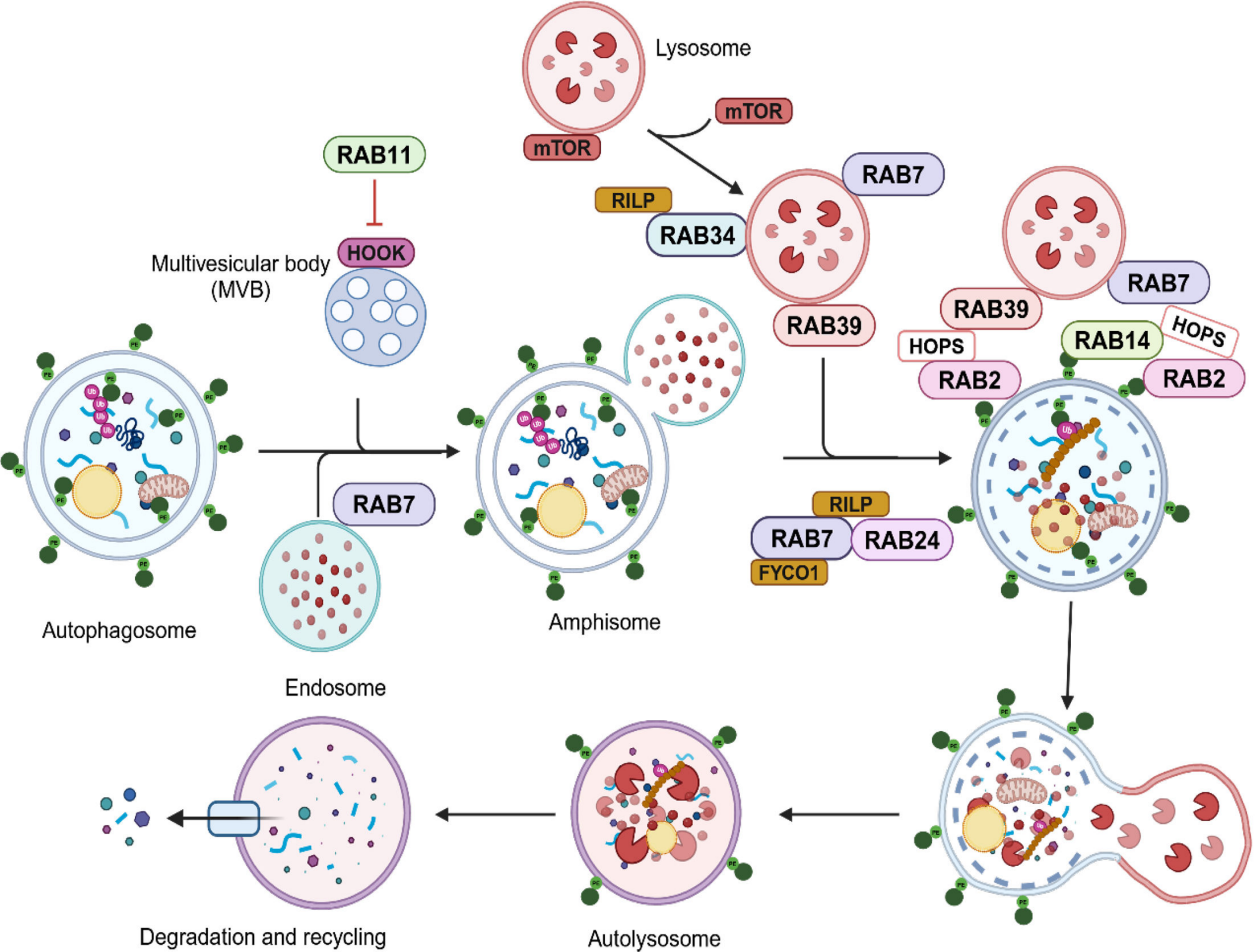
RAB proteins, including RAB7, RAB24, RAB27, and others, facilitate the recruitment and fusion of autophagosomes and lysosomes in the perinuclear region to form autolysosomes. This process triggers cargo degradation, nutrient recycling, and mTOR reactivation, leading to completion of the autophagic cycle.

Likewise, RAB11 has also been involved in both autophagosome biogenesis and maturation. Initially, our laboratory presented evidence that RAB11 was required for docking and fusion of multivesicular bodies (MVBs) [63], and we demonstrated that RAB11 is involved in the fusion of MVBs with autophagic vesicles necessary for their maturation in amphisomes [64], while others showed that RAB11 depletion leads to a reduction in the tethering of LC3-decorated vesicles with RAB11-positive MVBs (see Figure 3) [65]. In *D. melanogaster,* it was demonstrated that RAB11, together with the Drosophila homolog of sorting nexin 18, regulates trafficking from REs to the autophagosome formation sites as a membrane source for phagophore expansion [65]. Work from the laboratory of S. Tooze and collaborators points to RAB11 recycling endosomes as participants in autophagosome biogenesis [66]. More recently, it has been shown that recycling endosomes are key for the biogenesis of the initial autophagosomal structures by the recruitment of basic components of the autophagy machinery, such as WIPI2 and subsequently ATG16L1, contributing to determining the sites for LC3 conjugation [64]. RAB26 has also been connected to regulating the recruitment of ATG16L1 onto pre-autophagosomal compartments participating in the autophagic clearance of synaptic vesicles in neurons [67]. RAB13 appears to be a positive regulator of autophagy in vascular endothelial cells since its depletion leads to a decrease at the transcriptional level of critical ATG genes such as ULK1 and ATG16L1 [68].

The ER-associated RAB32 has been implicated in supplying membranes from ER for the generation of autophagosomes, since overexpression of wildtype or an active RAB32 mutant induces autophagosome formation even under nutrient-rich conditions [69]. In addition, RAB32 promotes the autophagic degradation of mitochondrial-proximal ER membranes [70]. Another important autophagy player is the Golgi-associated RAB33, in particular the isoform RAB33B, which induces lipidation of LC3 and accumulation of autophagosomes even under nutrient-rich conditions [71]. This GTPase was the first RAB protein to be shown to interact with components of the ATG machinery, specifically with ATG16L1 [41,72]. As depicted in Figure 2, RAB33B binding to ATG16L, which is GTP-dependent, facilitates the recruitment of the complex formed by this protein with the ATG12-ATG5 conjugate onto the phagophore [41,71,72].

RAB16 also appears to be involved in the early steps of autophagosome formation, participating in the recruitment of membranes for the phagophore elongation. It has been proposed that RAB16 may associate with other RABs such as RAB5 and RAB7 that are involved in autophagy [36].

A recent publication has demonstrated that RAB21 plays a crucial role in the secretion of ATP via fusion of autophagic compartments (i.e. amphisomes) with the plasma membrane. Of note, KO and overexpression experiments indicate that RAB21 also plays a positive role in autophagosome biogenesis, suggesting also a function in the early steps of autophagosome formation [73].

Likewise, the small GTPase RAB17, which is mainly associated with recycling endosomes, contributes to the supply of membranes, proteins, and lipids required for the biogenesis and completion of autophagosomes [74]. It was shown that these membranes also contribute well to the generation of the vacuoles that entrap the bacteria group A *Streptococcus* (GAS). RAB17 colocalizes GAS-containing autophagosome-like vacuoles (GcAVs), and experimental data suggest the involvement of RAB17 in the fusion of recycling endosomes with the GcAVs. Thus, RAB17 participates in the antibacterial autophagic response against GAS [74].

This suggests that RAB14 is likely involved in the early stages of autophagosome formation. Furthermore, RAB14 directly interacts with KLP8A, which is localized on the surface of LC3-positive autophagic vesicles. This interaction modulates both the size and positioning of these vesicles [41,75].

Studies in the worm *Caenorhabditis elegans* have shown that RAB10 is mainly involved in stimulating autophagic flux by facilitating the recruitment of ATG9A to autophagic vesicles [76]. Cells lacking RAB10 display an increased number of autophagosomes, like in cells with an autophagosome fusion defect with lysosomes. Thus, this GTPase plays an important role in autophagy, in both the regulation of autophagosome formation and fusion [76].

In GAS infections, it has been observed that RAB7 is crucial in forming LC3-bound vacuoles containing GAS. Additionally, it was determined that RAB7 recruits ATG5-marked membranes in GAS-infected cells, but not in uninfected cells [77]. This suggests that RAB7 is involved in the early stages of autophagy in response to pathogens but not in canonical autophagy [77].

It has been suggested that the RAB3 GAP RAB3GAP1/2 participates in autophagosome biogenesis by analyzing ATG5 puncta generation upon treatment with rapamycin, an mTORC1 inhibitor. RAB3GAP1/2 localizes with LC3 to lipid droplets, and depletion of this GAP causes a decrease in the lipidation of the member of the LC3 protein family [78,79]. However, these effects are independent of RAB3, indicating that another undefined RAB GTPase might interact with RAB3GAP1/2 in this context [78]. Thus, so far, there are no indications that RAB3 is involved in autophagy.

### RABs involved in the final stages of autophagy: the maturation step

Several RAB proteins participate as regulators of crucial processes such as autophagosome maturation and fusion with lysosomes, leading to cargo degradation. Working in concert with their specialized effectors, tethering complexes, SNARE proteins, and motor proteins, RABs ensure the efficient maturation and fusion of autophagosomes with lysosomes. Defects in these systems can cause autophagic dysfunction, leading to diseases such as neurodegeneration and cancer. Some RAB proteins that have been linked to these processes are listed below.

RAB7 is a master regulator of autophagy by mediating fusion with lysosomes [79–82]. In yeast mutants lacking Ypt7, the RAB7 ortholog accumulates autophagic vesicles unable to fuse with lysosomes [83,84]. RAB7 interacts with different effector molecules such as FYCO1 (FYVE and coiled-coil domain autophagy adaptor 1) and Rab-interacting lysosomal protein (RILP) to allow transport of autophagosomes via microtubules toward their positive and negative ends, facilitating their transport to the cell periphery and perinuclear region, respectively [85]. The activation of RAB7 by the Monensin sensitivity protein 1-caffeine, calcium, and zinc 1 complex (Mon1-Ccz1 complex) leads to the recruitment of the membrane tethering homotypic fusion and protein sorting (HOPS) complex to the surface of autophagosomes. The VPS41 subunit of the HOPS complex interacts with activated RAB7 located on the lysosomal membrane, facilitating fusion with the autophagosome and enabling the formation of the autolysosome (see Figures 1–3) [86,87]. As depicted in Figure 3, RAB34 also interacts with its effector RILP to orchestrate the perinuclear positioning of lysosomes, a critical process for maintaining efficient autophagic flux [88].

Studies in *Drosophila* have revealed that RAB2 localizes to autophagosomal membranes, where it also interacts with the HOPS complex, likely facilitating the fusion of amphisomes with RAB7-labeled structures, probably late endosomes and lysosomes [89]. Recent studies have demonstrated that RAB14 is phylogenetically similar to RAB2 [90,91]. Knockout cells for RAB2 and RAB14 have LC3 accumulation during both normal autophagy and autophagy triggered by starvation, which means a decrease in the autophagic flux. Additionally, the involvement of both RABs in the recruitment of the HOPS complex to the surface of autophagosomes has been demonstrated. This recruitment is essential for the fusion of autophagosomes with lysosomes, a critical step for the continuation of autophagic flux [91].

It has been shown that RAB19 binds to the HOPS complex as well; thus, it is required for maintaining a proper autophagic flux. Inhibition of this interaction leads to the accumulation of autophagosomes and a block in autophagy, indicating that this GTPase is indeed required to maintain a proper autophagic flux [92].

Several reports have revealed that RAB24 plays an important role in the maturation of the autophagic compartment. We have demonstrated that under nutrient-rich conditions, RAB24 forms a complex with RAB7 and RILP, which is essential for the late endosomal/lysosomal degradation process by facilitating the transport of late endosomes and lysosomes along microtubules (see Figure 3) [93]. Additionally, we showed that following starvation-induced autophagy, there is a significant change in the subcellular distribution of RAB24, transitioning from a peripheral ER-like distribution to a vesicular punctate pattern [94]. A large percentage of the RAB24-labeled puncta colocalized with LC3 puncta, indicating that those could be autophagosomes [94]. In a subsequent study, we revealed that upon glucose deprivation, an inducer of autophagic response also leads to the formation of aggresomes in primary cultured cardiac myocytes [95]. These aggresomes colocalized with GFP-LC3 and RAB24, and a marked increase in RAB24- and ubiquitin-labeled puncta was observed after exposure to glucose deprivation. Later, it was proved that RAB24 is crucial for the clearance of mature autophagic vacuoles under basal conditions, being essential for the degradation of autolysosomes under nutrient-rich conditions [96]. In addition, RAB24 also facilitates the clearance of mutant huntingtin aggregates in HeLa cells [96]. Importantly, mutations in RAB24 have been associated with a few diseases, including ataxia and cancer, but it remains to be established if the diseases are caused by a defect in autophagy [97].

In a recent publication, it was demonstrated that RAB39A mediates the fusion of autophagosomes with lysosomes, since a significant reduction in autophagic flux in RAB39A knockout cells [98]. Mechanistically, RAB39A has been identified as a key small GTPase that recruits HOPS onto autophagic vesicles, a complex that mediates tethering of membranes and is known to participate in the fusion of autophagosomes with lysosomes (see Figure 3) [98,99].

RAB9 localizes to late endosomes, and it is required in the maintenance of the late endosomal compartments and for vesicular traffic from these organelles to the trans-Golgi network [100,101]. RAB9 is also present on autolysosomes, and in addition, it seems to be involved in the generation of autophagosomes from the TGN in an alternative autophagy mechanism independent of ATG5 and ATG7 [102,103].

RAB20 localizes to autophagic/phagosomal vesicles and is recruited during their maturation process. It appears to be required for the efficient fusion of autophagosomes with lysosomes, but this still needs further investigation to assign a specific function to RAB20 in autophagy [104,105]. RAB22 interacts with early endocytic and phagocytic compartments, specifically binding to early endosome antigen 1 in its GTP-bound form. This interaction promotes the maturation of these compartments, a process that is essential for the optimal progression of autophagy, particularly under cellular stress conditions [106]. In PC12 cells, RAB22 overexpression and nerve growth factor (NGF) deprivation positively altered autophagy progression. It has been demonstrated that RAB22, along with its effector Rabaptin-5-associated exchange factor for RAB5, is necessary for endosome biogenesis and NGF signaling [107,108]. In addition, intracellular bacteria such as *M. tuberculosis* and *Staphylococcus aureus* exploit RAB22 to create their replicative vacuole by modulating autophagy [105]. Likewise, the Golgi resident RAB30 has been involved in the generation of GcAVs, the compartment in which this bacterium resides and multiplies [109]. Similarly, RAB23 may also play a role in the subversion of autophagy by bacteria since RAB23 is important for GAS targeting the autophagic vacuoles (autophagosomes) to generate its intracellular niche [110]. It appears that RAB15 negatively regulates autophagy since its depletion promotes the maturation of early endosomes into late endosomes, which in turn will fuse with degradative lysosomes [111]. In a very recent publication, it has been shown that in cells infected with *Ehrlichia chaffeensis*, a bacterium from the rickettsiae family, autophagy is induced, and the pathogen generates vacuoles with early endosomal characteristics, which do not fuse with lysosomes. Interestingly, this pathogen up-regulates RAB15, likely to evade autophagic degradation [111].

### RABs with other autophagy-related functions

The function of RAB8 in autophagy is not well known [38]. RAB8B seems to regulate autophagy via its downstream effector TANK-binding kinase 1 (TBK1), a Ser/Thr kinase involved in selective types of autophagy through phosphorylation of the SARs [32]. On this line, RAB8B, also via TBK1, has been involved in autophagy-mediated antimicrobial activity against *Mycobacterium tuberculosis* var. *bovis* BCG, allowing the maturation of autophagosomes into degradative organelles [112]. On the one hand, RAB18 modulates lipid droplet metabolism and facilitates the channeling of lipids derived from lipid droplets into the biogenesis of autophagosomes by ATG9 [113]. However, it has also been shown that RAB18 participates in maintaining cellular homeostasis in hepatic stellate cells, and its depletion knockdown increases autophagy flux and partly inhibits proteostasis [114].

It has recently been shown that RAB14 promotes epithelial to mesenchymal transition in bladder cancer via the influence of the PI3K/AKT signaling pathway [115]. In addition, it has been reported that a serine–threonine protein kinase from *M. tuberculosis*, the eukaryotic-type protein kinase G (PknG), inhibits autophagy by inhibiting RAB14 GTP hydrolysis, and this prevents autophagosome maturation, blocking autophagy. Thus, this result indicates that RAB14 is also involved in mediating fusion of autophagosomes with lysosomes, regulating the autophagic flux [116].

The role of RAB25 in autophagy is associated with specific types of tumors, such as ovarian and breast cancers [117,118]. Silencing of RAB25 activates autophagy and negatively regulates the cell cycle, inhibiting cell growth and inducing apoptosis in ovarian tumor cells [119]. In contrast, RAB26 expression levels are reduced in aggressive cancer cell lines, suggesting that activation of RAB26-autophagy-dependent pathways may prevent metastasis and tumor progression [120].

Regarding RAB27**,** recent studies have highlighted the functionality of its isoform RAB27B in the fusion of autophagosomes with lysosomes, contributing to the clearance of α-synuclein, a constituent of the proteinaceous aggregates termed Lewy bodies that accumulate in the neurons of Parkinson’s disease patients [121]. RAB27B has also been found to be a regulator of proliferation and survival of colorectal cancer cells by enhancing autophagic flux [122]. Likewise, alterations in RAB29 functionality disrupt autophagic flux through multiple mechanisms. RAB29 recruits and activates leucine-rich repeat kinase 2, which interacts with key autophagic effectors such as p62, LC3, and BECN1. RAB29 dysfunction impairs autophagosome transport, maturation, and lysosomal function. These perturbations collectively contribute to the accumulation of toxic protein aggregates associated with neurodegenerative disorders like Parkinson’s disease [123,124].

RAB21 has been shown to be an indirect regulator of autophagy through its role in the retromer-dependent recycling of transmembrane proteins such as the glucose transporter GLUT1 at endosomes [125]. GLUT1 is critical for maintaining cellular energy homeostasis, and depletion of RAB21 leads to a missorting and degradation of GLUT1 in lysosomes. In turn, this causes an alteration in glucose uptake by the cells, leading to the activation of the AMPK and thus an induction of the autophagic flux [125].

**Figure 4:**
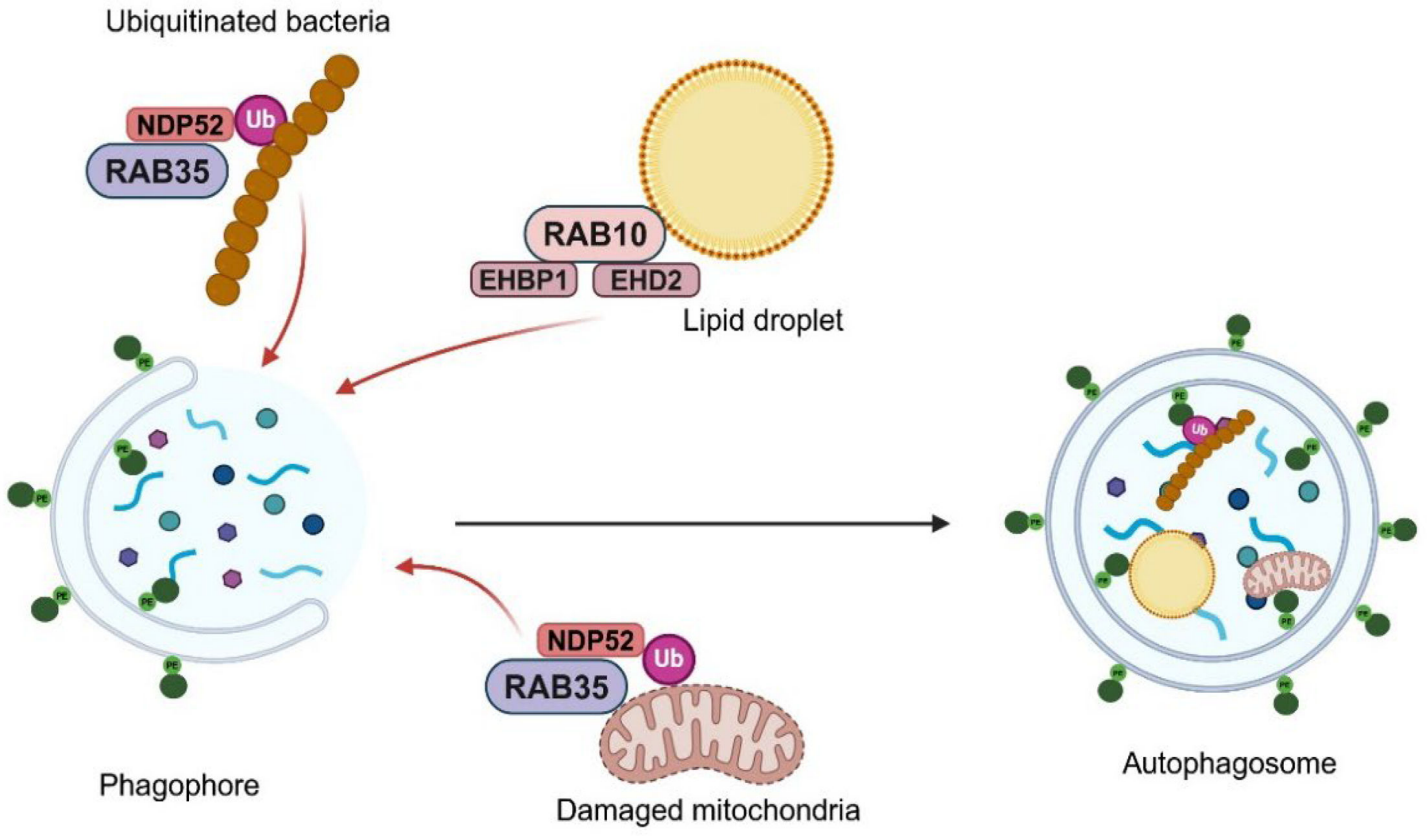
RAB proteins, including RAB10, RAB35, and others, are crucial for cargo sequestration in autophagosomes. They co-ordinate the recruitment of ubiquitinated bacteria, damaged mitochondria, and lipid droplets, ensuring efficient phagophore expansion and autophagosome maturation.

RAB10 also participates in the modulation of lipid metabolism by enhancing lipophagy (degradation of lipid droplets). Indeed, RAB10 is located on the surface of lipid droplets and recruits proteins such as EHBP1 and EHD2, forming a complex that promotes the extension of the phagophore along the lipid droplet (see Figure 4) [126]. In the case of RAB35, this GTPase interacts with autophagy receptors such as NDP52, which are essential for directing ubiquitinated cargo, such as bacteria and damaged mitochondria, towards autophagic degradation. This interaction is fundamental for the efficient removal of damaged cellular components and pathogens (see Figure 4) [127].

## Final remarks

We have presented a comprehensive review about the RAB GTPases known to be involved in autophagy. Numerous RABs, including RAB1, RAB4, RAB5, RAB9, RAB13, RAB16, RAB18, and RAB32 (see Figure 2), have been functionally connected to the initial steps of this pathway, whereas others such as RAB2, RAB7, RAB24, and RAB39 (Figure 3) appear to be only involved in regulating maturation and fusion of autophagosomes. In addition, some RABs, such as RAB11, RAB14, and RAB33B, may have a dual protagonist function because they are playing a key role in both autophagosome formation and maturation.

There is limited information about the role of other RAB proteins in autophagy and for this reason, they have not been mentioned in this review. However, it may be predictable that additional RABs will be associated with different autophagy steps, providing crucial information about the mechanism of autophagy, especially when effectors are identified.

## Open Access

This article has been published open access under our Subscribe to Open programme, made possible through the support of our subscribing institutions, learn more here: https://portlandpress.com/pages/open_access_options_and_prices#conditional

## Abbreviations

AMPK: adenosine monophosphate-activated protein kinase
ATGs: autophagy-related genes
DFCP1: double-FYVE containing protein 1
ER: endoplasmic reticulum
ER: endoplasmic reticulum
GAPs: GTPase-activating proteins
GEFs: guanine nucleotide exchange factors
GcAVs: GAS-containing autophagosome-like vacuoles
HOPS: homotypic fusion and protein sorting
MVBs: multivesicular bodies
NDP52: nuclear dot protein 52 kDa
NGF: nerve growth factor
PAS: pre-autophagosomal structure
PAT4: proton-coupled amino acid transporter 4
PI3KC3-C1: class III phosphatidylinositol 3-kinase complex I
PI3P: phosphatidylinositol 3-phosphate
PtdIns3P: phosphatidylinositol 3-phosphate
RAB: RAS related in brain
RILP: Rab-interacting lysosomal protein
SARs: selective autophagy receptors
SNAREs: soluble NSF attachment protein receptors proteins
TBK1: TANK-binding kinase 1
TGN: Trans-Golgi Network
ULK: Unc-51 like autophagy-activating kinase
mTORC1: mechanistic target of rapamycin complex 1
